# Organizing the Vertebrate Embryo—A Balance of Induction and Competence

**DOI:** 10.1371/journal.pbio.0020127

**Published:** 2004-05-11

**Authors:** Igor B Dawid

## Abstract

The current understanding of organizer formation and neural induction in vertebrate embryos is discussed

In what is usually referred to as the most famous experiment in embryology, Hans Spemann and Hilde Mangold (1924) showed that a specific region in early frog embryos called the blastopore lip can induce a second complete embryonic axis, including the head, when transplanted to a host embryo. Most of the axis, including the nervous system, was derived from the host, whose cells were induced to form an axis by the graft, therefore named the organizer. Induction refers to the change in fate of a group of cells in response to signals from other cells. The signal-receiving cells must be capable of responding, a property termed competence. The Spemann–Mangold organizer. which—as the transplantation experiment shows—is able to turn cells whose original fate would be gut or ventral epidermis into brain or somites, is the prototypical inducing tissue. And neural induction has for a long time been regarded as a process by which organizer signals, in their normal context, redirect ectodermal cells from an epidermal towards a neural fate. The nature of the neural inducer or inducers and the mechanism of neural induction have been and remain hot topics in developmental biology.

For half a century after Spemann and Mangold, studies on amphibians monopolized the subject, and even more recently, a large part of the progress in analyzing organizer formation and function and neural induction was based on amphibians, mostly the model species Xenopus laevis. In the past few years, however, work in other vertebrate and nonvertebrate chordate systems has come to play an important role in the field and has shed light on generalities and differences among chordates. If the present primer uses Xenopus to illustrate the process, it is because it accompanies an article in this issue of *PLoS Biology* dealing with neural development in this species (Kuroda et al. 2004) and, of course, because of the experience of this author. Here I shall outline the understanding of organizer formation and neural induction as it has evolved over recent times and attempt to integrate recent results from different species into a common pattern.

## Cortical Rotation and Nuclear Localization of β-Catenin

The frog egg is radially symmetrical around the animal–vegetal axis that has been established during oogenesis. Fertilization triggers a rotation of the cortex relative to the cytoplasm that is associated with the movement of dorsal determinants from the vegetal pole to the future dorsal region of the embryo (Gerhart et al. 1989). (A brief parenthetical point is in order here. Conventionally, the side of the amphibian and fish embryo where the organizer forms has been called dorsal, with the opposite side labeled as ventral. This axis assignment does not project unambiguously onto the clearly defined dorsal–ventral polarity of the larva, as pointed out forcefully in recent publications [Lane and Smith 1999; Lane and Sheets 2000, 2002]. In these papers, a new proposal is made for polarity assignments in the gastrula that, I believe, has some merit, but also presents some difficulties. As the conventional approach of equating organizer side with dorsal seems to remain in wide use at present, I shall apply this convention, albeit with the reservation above.)

**Figure 1:**
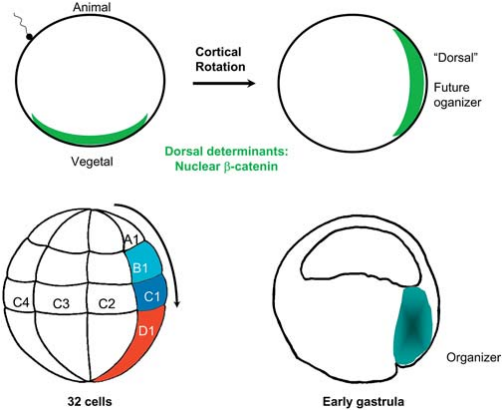
Early Development in X. laevis After fertilization, dorsal determinants are transported from the vegetal pole to one side of the embryo, where β-catenin will achieve nuclear localization. By 32 cells, the row of cells labeled 1 is specified as dorsal. Movements towards the vegetal pole (arrow) start at early cleavage stages. The organizer forms from C1 and B1 progenitors, the dorsal ectoderm or BCNE mostly from A1 progenitors (see Figure 2). The organizer is indicated in the gastrula embryo. See the text for further explanation.

While the nature of the dorsal determinants is still in dispute, it is clear that the consequence of their translocation is the nuclear localization of β-catenin in a wide arc at the future organizer side (Figure 1) (Schneider et al. 1996; Schohl and Fagotto 2002). Nuclear localization of β-catenin appears to be the first event that determines dorsal/ventral polarity in the Xenopus and zebrafish embryos (Hibi et al. 2002). No comparable early event appears to be involved in amniote (e.g., chick and mouse) embryos.

## Induction by the Organizer: Antagonizing Bone Morphogenetic Protein

As gastrulation starts, the Spemann–Mangold organizer, which includes mostly axial mesodermal precursors, was classically believed to instruct naïve ectoderm to convert to neural tissue. In transplant or explant studies, animal ectoderm that forms epidermis, when undisturbed, is susceptible to neural induction by the organizer. This fact prompted a search for neural inducers that eventually led to the identification of several substances with the expected properties—organizer products that can neuralize ectoderm. Their molecular properties were at first surprising: they proved to be antagonists of other signaling factors, mostly of bone morphogenetic proteins (BMPs) and also of WNT (a secreted protein homologous to the Drosophila Wingless protein) and Nodal factors (Sasai and De Robertis 1997; Hibi et al. 2002). These observations led to the formulation of a “default” model of neural induction (Weinstein and Hemmati-Brivanlou 1997), which states that ectodermal cells will differentiate along a neural pathway unless induced to a different fate. The heuristic simplicity and logical cogency of this model facilitated its wide acceptance, although it did not explain the processes that set the “default.” Some of these processes have been the subject of subsequent studies that were conducted in several different species, and this has led to a more refined (and probably more accurate) picture.

## The Role of Fibroblast Growth Factor

For example, additional signaling pathways are now known to operate. Recent work on neural induction comes to two major conclusions: (i) the fibroblast growth factor (FGF) signaling pathway plays a major role in this process, and (ii) neural specification starts well before gastrulation and thus before the formation and function of the organizer. Studies on the role of FGF in early Xenopus development initially discovered its role in mesoderm induction and the formation of posterior tissues (Kimelman et al. 1992). And while the involvement of FGF in neuralization was observed early in this system (Lamb and Harland 1995; Launay et al. 1996; Hongo et al. 1999; Hardcastle et al. 2000), in view of the impressive effects seen with Chordin and other BMP pathway antagonists, the relevance of FGF in neural specification in amphibians and fish was slow to be recognized. It took elegant studies, mostly in chick embryos (Streit et al. 2000), and their eloquent exposition (Streit and Stern 1999; Wilson and Edlund 2001; Stern 2002) to turn the tide, but there is now no doubt that the FGF signaling pathway plays a major role in the specification and early development of the neural ectoderm in chordates.

FGF does not seem to behave as a classical organizer-derived neural inducer, however. Maternal FGF mRNA and protein appear to be widely distributed in the early embryo, and at least one FGF family member is expressed primarily in the animal, pre-ectodermal region during blastula stages (Song and Slack 1996). A detailed study of the regions where different signaling pathways are active during embryogenesis (Schohl and Fagotto 2002) showed that the entire ectoderm is probably exposed to FGF signals at or prior to the time of neural induction, with the more vegetal, mesoderm-proximal region of the ectoderm being exposed to higher levels. Thus, exposure to FGF is required to endow the ectoderm with the competence to respond to additional signals that will act later on its way towards neural specification. Such a process was deduced from experiments in the chick, where an FGF signal must be followed by exposure to organizer signals to sensitize the tissue to BMP antagonists that ultimately stabilize the neural fate (Stern 2002).

An exciting recent study shows that exposure of the epiblast (ectoderm) to FGF induces, after a time delay, a transcription factor named Churchill. Churchill expression inhibits cell ingression leading to mesoderm formation; the cells remaining in the epiblast assume a neural fate (Sheng et al. 2003). The time delay in Churchill induction appears to be the key in explaining how one signal, FGF, can be involved in mesodermal and neural development at the same time in cells that are in close proximity. The question how FGF signaling can lead to different outcomes was also addressed in a study on neural specification in ascidians (Bertrand et al. 2003). Here, the FGF signal leads to neural induction through the coordinated activation of two transcription factors, Ets1/2 and GATAa, whereas FGF does not activate GATAa during its function in mesoderm formation. Thus, similar input leads to distinct output as a result of different responses by target tissues, stressing the importance of competence in this inductive process.

## Molecular Predisposition

**Figure 2:**
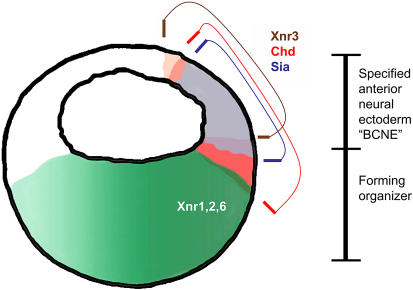
Expression Patterns in Dorsal Ectoderm Expression patterns of selected genes in the late blastula of Xenopus, based on the work of Kuroda et al. (2004). See the text for further explanation.

Not surprisingly, then, attention has turned to the target tissues and to the prepatterns that might already exist. In Xenopus, it was long known that the animal region or pre-ectoderm is not uniform or naïve, in that the dorsal, organizer-proximal region is predisposed towards a neural fate (Sharpe et al. 1987). The paper by Kuroda et al. (2004) adds much information about neural specification before gastrulation in Xenopus and the factors involved in this process. The authors identify a region in the dorsal ectoderm of the blastula that they name the “blastula Chordin- and Nogginexpressing” (or BCNE) region (Figure 2). They show that this region, which I prefer to simply call dorsal ectoderm, expresses *siamois*, *chordin*, and *Xnr3*, another β-catenin target. The dorsal ectoderm or BCNE is fully specified as anterior neural ectoderm, as excision of this region led to headless embryos, and explants differentiated into neural tissue in culture, even when the formation of any mesodermal cells was blocked by interference with nodal signaling (Kuroda et al. 2004).

Kuroda et al. (2004) further show that induction of anterior neural tissue initiated by β-catenin requires Chordin, whereas formation of posterior neural tissue does not. This latter point concerns an issue not yet mentioned here, namely anterior–posterior patterning of the neural ectoderm, a process that occurs in concert with neural induction per se. This patterning appears to involve the interaction of various signaling factors, including FGF, BMP, WNT, and retinoic acid, all of which act as posteriorizing factors (Kudoh et al. 2002). Suppression of BMP signaling by expression of its antagonists is the condition that specifies the dorsal ectoderm or BCNE as future anterior neural ectoderm; in contrast, posterior neural ectoderm may form under the influence of FGF even in the presence of BMP signaling. The work by Kuroda et al. (2004) thus shows that initial specification of anterior neural ectoderm in Xenopus, as in other vertebrates, takes place before gastrulation and does not require organizer signals; this is not to say that full differentiation and patterning of the nervous system could be achieved without organizer participation.

## Induction *and* Competence

The formation of the vertebrate nervous system thus depends on multiple signaling pathways, such as the FGF, BMP, and WNT signaling cascades, that interact in complex ways (e.g., Pera et al. 2003). In contrast to the classical view, neural induction is not exclusively promoted by organizer-derived signals, in that earlier signals and intrinsic processes that determine ectodermal competence are prominently involved. Whether inductive signals or competence of responding tissue is more important in embryology has been debated, much like the nature–nurture controversy in the behavioral arena. Current work has given some boost to the competence side of the argument, but, as in behavior, the truth lies somewhere in between, though not necessarily at the halfway mark. Studies such as those discussed here bring us closer to finding the answer to this question.

## Abbreviations

BCNE region: blastula Chordin- and Noggin-expressing region
BMP: bone morphogenetic protein
FGF: fibroblast growth factor
